# Extensive set of African ancestry-informative markers (AIMs) to study ancestry and population health

**DOI:** 10.3389/fgene.2023.1061781

**Published:** 2023-02-23

**Authors:** Samantha Boudeau, Meganathan P. Ramakodi, Yan Zhou, Jeffrey C. Liu, Camille Ragin, Rob J. Kulathinal

**Affiliations:** ^1^ Department of Biology, Temple University, Philadelphia, PA, United States; ^2^ Cancer Prevention and Control Program, Fox Chase Cancer Center, Philadelphia, PA, United States; ^3^ African Caribbean Cancer Consortium, Fox Chase Cancer Center, Philadelphia, PA, United States; ^4^ Department of Biostatistics and Bioinformatics, Fox Chase Cancer Center, Philadelphia, PA, United States; ^5^ Department of Otolaryngology, Lewis Katz School of Medicine at Temple University, Philadelphia, PA, United States; ^6^ Department of Surgical Oncology, Fox chase Cancer center, Philadelphia, PA, United States

**Keywords:** African ancestry, aims, population structure, 1000 genomes project, ancestry and health

## Abstract

**Introduction:** Human populations are often highly structured due to differences in genetic ancestry among groups, posing difficulties in associating genes with diseases. Ancestry-informative markers (AIMs) aid in the detection of population stratification and provide an alternative approach to map population-specific alleles to disease. Here, we identify and characterize a novel set of African AIMs that separate populations of African ancestry from other global populations including those of European ancestry.

**Methods:** Using data from the 1000 Genomes Project, highly informative SNP markers from five African subpopulations were selected based on estimates of informativeness (In) and compared against the European population to generate a final set of 46,737 African ancestry-informative markers (AIMs). The AIMs identified were validated using an independent set and functionally annotated using tools like SIFT, PolyPhen. They were also investigated for representation of commonly used SNP arrays.

**Results:** This set of African AIMs effectively separates populations of African ancestry from other global populations and further identifies substructure between populations of African ancestry. When a subset of these AIMs was studied in an independent dataset, they differentiated people who self-identify as African American or Black from those who identify their ancestry as primarily European. Most of the AIMs were found to be in their intergenic and intronic regions with only 0.6% in the coding regions of the genome. Most of the commonly used SNP array investigated contained less than 10% of the AIMs.

**Discussion:** While several functional annotations of both coding and non-coding African AIMs are supported by the literature and linked these high-frequency African alleles to diseases in African populations, more effort is needed to map genes to diseases in these genetically diverse subpopulations. The relative dearth of these African AIMs on current genotyping platforms (the array with the highest fraction, llumina’s Omni 5, harbors less than a quarter of AIMs), further demonstrates a greater need to better represent historically understudied populations.

## Introduction

Racial health disparities in populations of African descent have been extensively documented and in the United States these disparities have been observed in many diseases (Tsai et al., 2011). For example, African Americans have significantly greater mortality and morbidity for asthma and are nearly five times more likely to be diagnosed with primary open-angle glaucoma compared to Americans with European ancestry (Barnes, 2010; Cole et al., 2021). Based on data from US cancer registries for all malignancies combined, African Americans have worse cancer incidence and survival rates compared with European Americans (Özdemir & Dotto, 2017). Not surprisingly, there are certain cancers for which the racial health disparity is more pronounced. In head and neck cancer, African Americans possess poorer survival rates than their European American counterparts even though they have a similar incidence rate (Daraei & Moore, 2015; Zavala et al., 2021). Breast cancer also has pronounced racial disparities with African American women having a 40% higher mortality rate, younger age at diagnosis, and higher incidence of aggressive forms of the disease. Interestingly, African American males also have a higher incidence of breast cancer compared to European American males (Stringer-Reasor et al., 2021). Compared to men of European ancestry, men of African or Afro-Caribbean ancestry have been found to have a higher risk of developing more aggressive forms of prostate cancer at a younger age (McHugh et al., 2021). Socioeconomic factors including healthcare access, geographical factors, lifestyle, and other behavioral factors are routinely used to explain racial health disparities in cancer (Khan et al., 2019). Yet, in head and neck cancer studies, much of this disparity remains after accounting for socioeconomic factors (Molina et al., 2008) and access to healthcare (Ragin et al., 2011), suggesting a genetic basis to these differences.

Much of the literature investigating racial health disparities has relied on self-identified race as a proxy for genetic ancestry. However, current characteristics for determining race, including skin color, geography, and language, are often too vague to capture true genetic ancestry in disease studies (Hunt and Megyesi, 2008). Ancestry-informative markers (AIMs) are SNPs with highly differing allele frequencies between different populations, and the differences in frequency tends to be an order of magnitude greater than the difference among continental subpopulations (Tian et al., 2006). More recently, AIMs are being integrated in biomedical studies to study associations between genetic ancestry and health as a more accurate measure of genetic ancestry. AIMs panels are heavily used in admixture mapping in studies seeking to identify disease-associated loci in admixed populations such as African Americans (Chen et al., 2010). AIMs allow for the study of both global and local ancestry association with disease which can lead to the identification of population-specific disease loci. Loci that are associated with increased disease risk in a population are likely to be found in regions of the genome with a high percentage of ancestry for that population (Zhang et al., 2014). While studies have shown that only 1,500–2,500 SNPs are necessary to detect ancestral chromosomal regions in admixed populations (Winkler et al., 2010), a comprehensive AIMs set is required for a finer mapping of disease loci.

In this work, we generate a novel panel of 46,437 African ancestry-informative markers that were identified using European and African subpopulations genotype data from Phase 3 of the 1000 Genomes Project (1KGP) (Sudmant et al., 2015). This work compares populations of African and European ancestries to identify SNPs that will be highly informative for African ancestry as well as differentiating Africans from other continental groups. Additionally, this AIM set will provide an important reference panel to investigate genetic ancestry in understudied admixed populations such as African Americans and Afro-Caribbeans. While most African AIMs found in the literature are comprised of SNPs from just two subpopulations, namely CEU (US-European) and YRI (Yoruba) (Zhang et al., 2014), this panel provides a more extensive accounting of each of the 1KGP subpopulations in Europe (EUR) and Africa (AFR). The most highly differentiated SNPs from each AFR subpopulation relative to the EUR population based on informativeness estimates common to all AFR subpopulations were pooled to generate this panel. The African AIMs were validated using an independent dataset of 1,448 individuals where approximately one half is of European ancestry and the other half is of varying degree of African ancestry. They were characterized for function and disease association with several gene-trait associations specific to African populations corroborated in the literature. We also find that these SNPs are under-represented in major genotyping platforms that are in current use. This work identifies a robust new panel of SNPs found in high frequency across continental African populations that have the potential to link population-specific mutations and disease, thus, providing much needed foundational data to examine understudied African populations.

## Materials and methods

### Data source and AIMs identification

**FIGURE 1:**
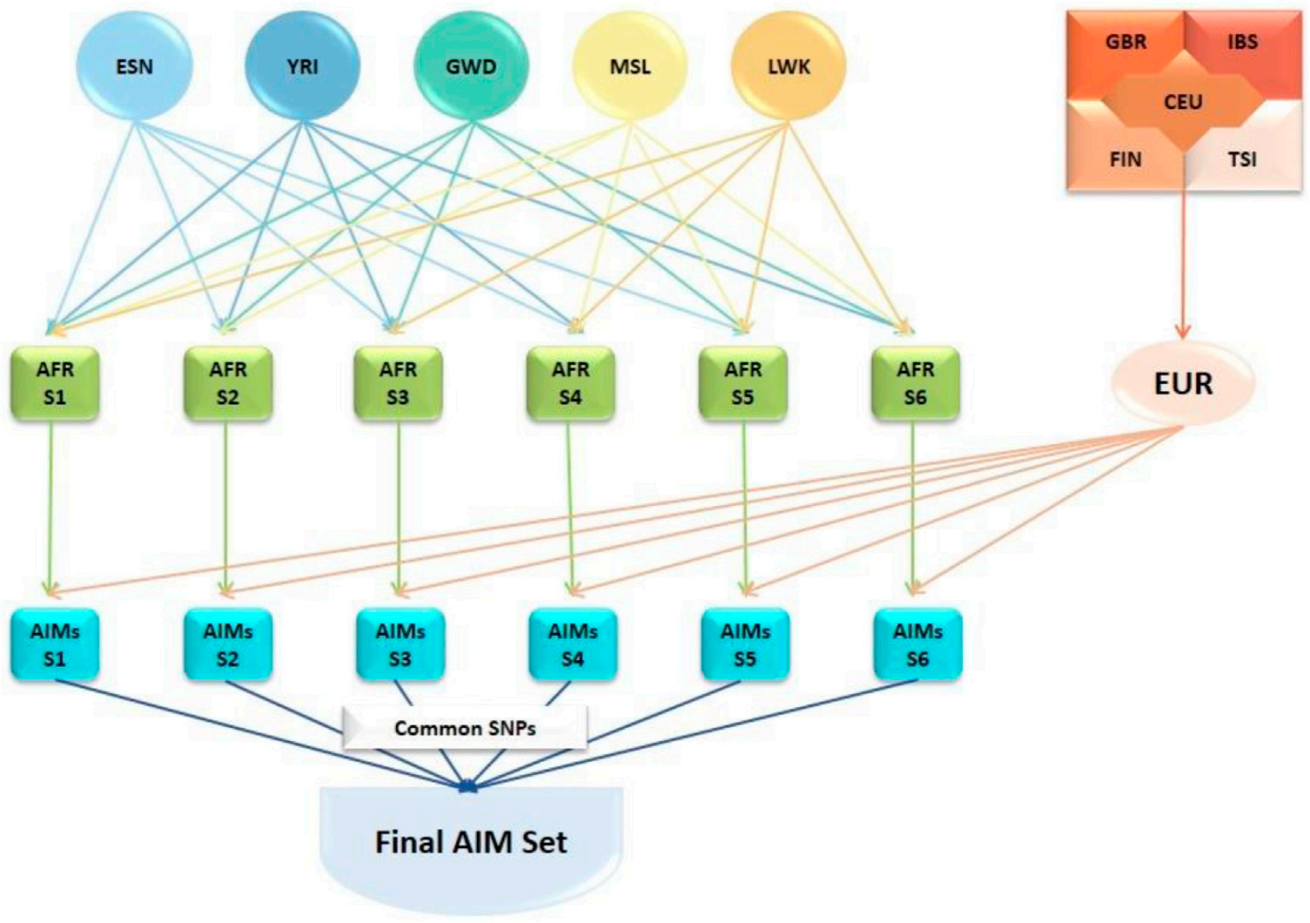
Development of the African AIMs panel for this study. Genotype data from African (AFR) populations (Gambian, Gambia [GWD]; Esan, Nigeria [ESN]; Luhya, Kenya [LWK]; Mende, Sierra Leone [MSL]; Yoruba, Nigeria [YRI]) and European (EUR) populations (Utah residents with European ancestry [CEU]; Finnish, Finland [FIN], British; England & Scotland [GBR]; Iberian, Spain [IBS]; Tuscany, Italy [TSI]) from the 1000 Genomes Project database. AFR dataset combinations (AFR-S1 to AFR-S6) and the combined EUR dataset were generated based on the ancestry informativeness (ln) of each variant. Genetic variants with *In*≥ 0.25 were considered as AIMs. Each AFR subset was compared against the combined EUR set resulting in six different AIMs subsets. The SNPs common to all six AIMs subset were extracted to generate the set of 46,737 African AIMs used in this study.

Ancestry-informative markers (AIMs) were identified using SNP data from the 1000 Genomes Project (1KGP) (1000 Genomes Project 2012; 1000 20151000 Genomes Project Consortium et al., 2015). The genotype data of African (AFR) populations (Gambian, Gambia [GWD]; Esan, Nigeria [ESN]; Luhya, Kenya [LWK]; Mende, Sierra Leone [MSL]; Yoruba, Nigeria [YRI]), and European (EUR) populations (Utah residents with European ancestry [CEU]; Finnish, Finland [FIN], British, England & Scotland [GBR]; Iberian, Spain [IBS]; Tuscany, Italy [TSI]) were downloaded from the 1KGP database (2013 release). The ancestry informativeness (*In*) of each genetic variant was estimated separately for six different combinations of AFR datasets (AFR-S1 to AFR-S6) and the combined EUR dataset (Figure 1) using the tool, infocalc (Rosenberg et al., 2003). Genetic variants with *In*≥ 0.25 were considered as AIMs. The analyses resulted in six separate AIMs subpanels which were subsequently intersected to identify 46,737 common African AIMs.

### Population genomic differentiation analysis

To validate population-specific properties of the identified AIMs, we estimated the pairwise fixation index (F_ST_) for the seven 1KGP subpopulations with significant African ancestry (YRI, ESN, MSL, GWD, LWK, ACB, ASW) against the European population. Using the 1KGP data for these populations, we extracted SNPs identified as AIMs on the 22 autosomes using BCFTools (Danecek et al., 2021). Weir-F_ST_ estimates were calculated using VCFTools (Danecek et al., 2011) and then visualized *via* violin plots.

We performed a principal component analysis on the AIMs set to visualize their ability to differentiate between the subpopulations of African ancestry and the other subpopulations from the 1KGP. Using BCFTools, the 1KGP VCF files were converted to their binary version (.bcf). PLINK was used to prune and merge the chromosome files into one. Eigenvectors were generated and used for PCA analysis comparing the different populations.

### AIMs validation

To test the ability of the identified AIMs to separate populations of African ancestry from those of European ancestry, we evaluated them on an independent dataset of 1,472 individuals. These individuals were recruited as part of a cohort of head and neck cancer study as cases and controls to be genotyped for an IRB-approved related study. A custom Illumina sequencing array was designed by adding a subset of the AIMs from this panel to the Illumina GSA backbone (Infinium Global Screening Array-24 Kit). DNA was extracted from the biospecimen collected from study participants as described in Blackman et al. (2018) and genotyped using the custom array. The AIMs included in the array are a subset of the 47 K that are not in linkage disequilibrium. After excluding retired SNPs, updated and combined SNPs, as well as SNPs whose probes were not able to be made, 11,377 probes were added to the GSA array. The number of AIMs successfully genotyped by all samples across two batches totaled 9,566 and the genotype data for 9,385 SNPs were used as the validation SNP subset *via* principal component analysis.

### Functional characterization

We annotated and evaluated the functional role of these genetic variants by running our list of AIMs through ANNOVAR (Wang et al., 2010) using the hg19 human reference genome. The pathogenicity of the AIMs was evaluated using multiple functional effect predictors: polymorphism phenotyping: PolyPhen2 (Adzhubei et al., 2013); sorting intolerant from tolerant: SIFT (Kumar et al., 2009); and two machine learning methods: metaSVM (support vector machine) and metaLR (logistic regression) (Dong et al., 2015).

### Overlap with current platforms

We also surveyed the representation of our AIMs set among common genotyping array platforms which may indicate how likely they are to be found in the literature as associated with known disease mutations. We downloaded the manifests for 25 commonly used genotyping arrays from Illumina and Affymetrix (ThermoFisher Scientific) and ascertained the number of SNPs from the AIMs set that appear on the sequencing arrays. Lastly, we estimated the fraction of our African AIMs represented on each array platform. These AIMs were then compared to a large list of imputed SNPs to estimate which of the AIMs could be imputed.

## Results

### Genomic characterization of African AIMs

**FIGURE 2:**
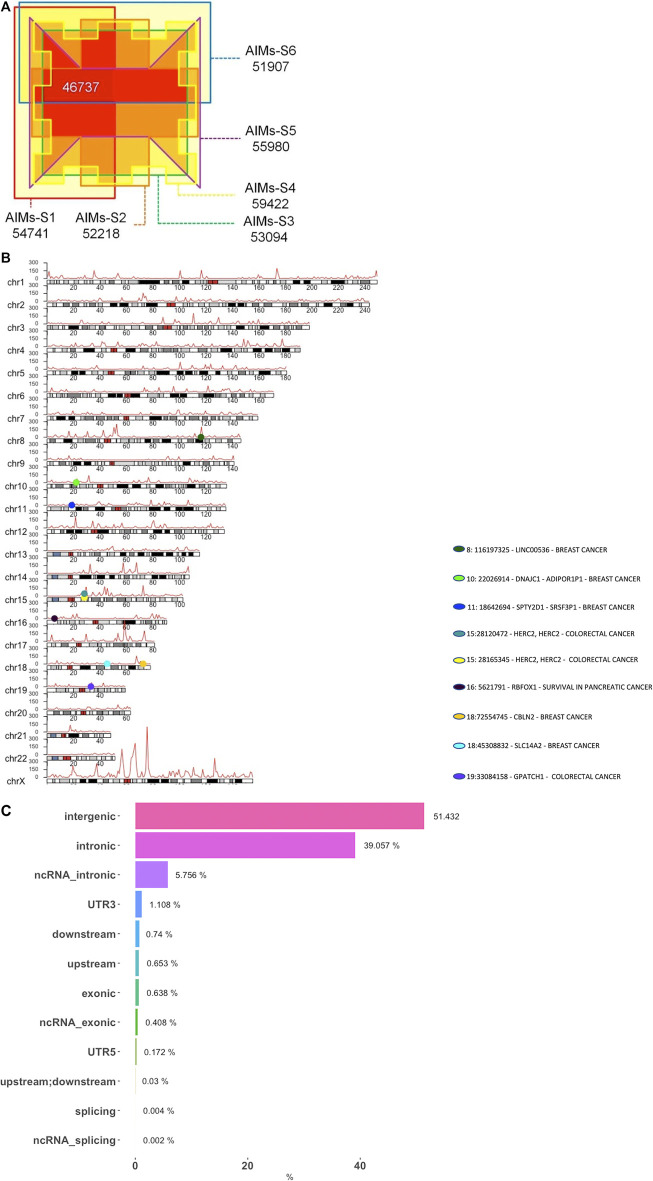
(Continued)

In this study, genotype data derived from five continental African and five European subpopulations were analyzed to obtain a generalized AIMs panel for the African populations represented in the 1KGP data (Figure 1). This African-European continental comparison initially generated six AIMs subpanels ranging from 51,907 to 59,422 SNPs with each subpanel including all African populations except for one. The number of AIMs obtained from each dataset are shown in Figure 2A. All six AIMs subpanels were intersected and a total of 46,737 AIMs commonly found in each of the six subpanels were retrieved for further analyses. The usefulness of these AIMs for assessment of population structure was analyzed using data from a separate and ongoing project pointing towards the utility of this AIMs panel to study population admixture in African populations (Ramakodi et al., 2017).

To characterize the distribution of these African AIMs in the genome and to evaluate their functional roles, we partitioned 1 Mbp windows across each chromosome. Figure 2B shows a genome-wide distribution of African AIMs with a significantly high fraction found on the X-chromosome. Most autosomal 1 Mbp windows have fewer than 300 SNPs, although there are several windows with a much higher SNP density. Chromosome 17 and the X-chromosome possess regions with a much higher density of SNPs, with some chromosomal regions having up to 970 SNPs per 1 MB window. Windows that are AIMs-rich are generally distributed evenly across each chromosome (e.g., chromosomes 1 and 12) with no obvious clusters. However, there appears to be some clusters of AIMs-rich windows, as is the case of the X-chromosome harboring a large cluster near its centromere. Additionally, some of the shorter chromosomes (e.g., chr8, 12, 14, 15, 17, and 20) appear to have a higher fraction of African AIMs relative to the longer chromosomes (e.g., chr2, 3, 5, 6, and 7).

To investigate whether the regions enriched for AIMs correlate with diseases in the literature, we extracted studies from the GWAS catalog that identified AIMs from this set as associated with cancer and plotted the loci (colored dots) onto a karyotype plot (Figure 2B). AIMs, rs12916300 and rs12913832, mapped to HERC2 (chr15) and rs694339 in the CBLN2 (chr18) genes were associated with colorectal cancer risk in a European sample (Hofer et al., 2017) (Figure 2B). In an African American sample, AIM rs7252505 found in the GPATCH1 gene (chr19) was associated with colorectal cancer risk (H. Wang et al., 2017) (Figure 2B). Two intronic AIMs (rs13267382: chr8, LINC00536; rs9952980: chr18, SLC14A2) and two intergenic AIMs (rs10832963: chr11, SPTY2D1 - SRSF3P1; rs11814448: chr10) were also found to be associated with breast cancer in samples of European and Asian ancestry (Michailidou et al., 2017).

Figure 2C shows the distribution of SNPs across different genomic regions and the potentially consequential roles they play in genome function. Most of the AIMs are found in two genomic regions, with just over half, 51.4%, found in the intergenic regions of the genome and 39.1% in intronic regions. The remaining SNPs are distributed in smaller fractions in intronic non-coding RNAs (5.8%), 3-prime UTRs (1.1%), with 0.7% found within 1 Mb downstream of genes and 0.6% found upstream of genes. Only 338 SNPs, 0.6% of the entire AIMs set, were found to be in coding regions of the genome. Interestingly, when compared to all autosomal SNPs in the 1000 Genomes Project dataset, this AIMs set has significantly fewer exonic (*p*-value < 2.2e-16, Fisher’s Exact test) and 5-prime UTR (*p*-value = 0.04685, Fisher’s Exact test) SNPs than expected.

### Substructure in populations of African ancestry

**FIGURE 3:**
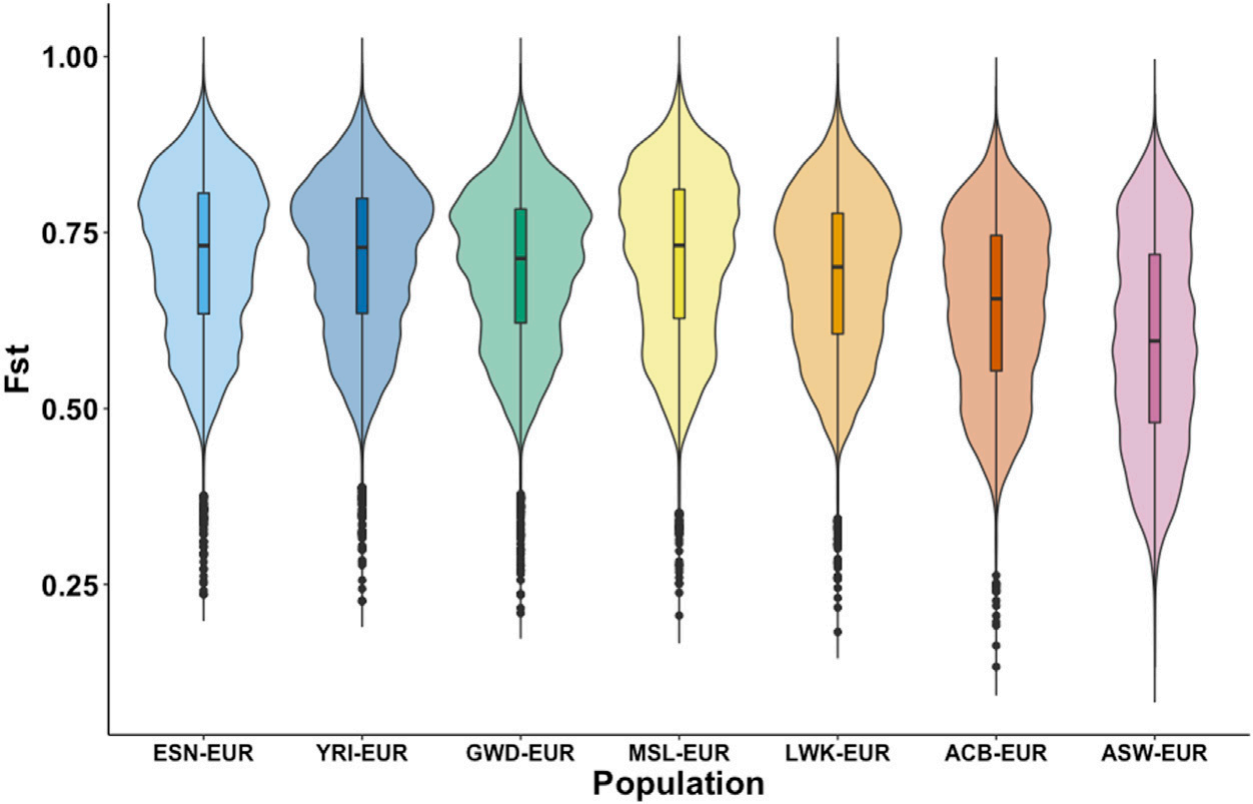
Distribution of FST among autosomal AIMs. FST was estimated for each African subpopulation (ESN, YRI, GWD, MSL, and LWK) against the European population using data from the 1000 Genomes Project. Additionally, estimates of FST were also calculated for the two other subpopulations in the dataset with high African ancestry, the Afro-Caribbean population from Barbados (ACB), and African Americans from the United States (ASW). FST was estimated for biallelic autosomal AIMs only and shows differences in the distribution in the FST estimates between the populations.

The importance of population substructure in association studies is becoming more apparent as they can confound observed genetic associations when ignored and hinder us from elucidating the genetic bases of disease. Consequently, understanding whether this set of African AIMs adequately identifies population substructure within and between populations with shared African ancestry is an important goal. Figure 3 shows the distribution of the fixation index of these AIMs in the different sub-populations of African ancestry as estimated against the EUR super-population. Based on Weir-F_ST_ statistics, there is a uniform distribution of F_ST_ estimates for the continental AFR subpopulations with the four West African subpopulations (ESN, YRI, GWD, MSL). The violin plots in Figure 3 show the most similarity. The ACB subpopulation shows a slightly different distribution compared to the AFR subpopulations though its plot still reflects a high degree of dissimilarity from the EUR population and close relationship to the AFR subpopulation. The ASW violin plot reveals the most divergent distribution and a much lower F_ST_ minimum relative to the other subpopulations.

**FIGURE 4:**
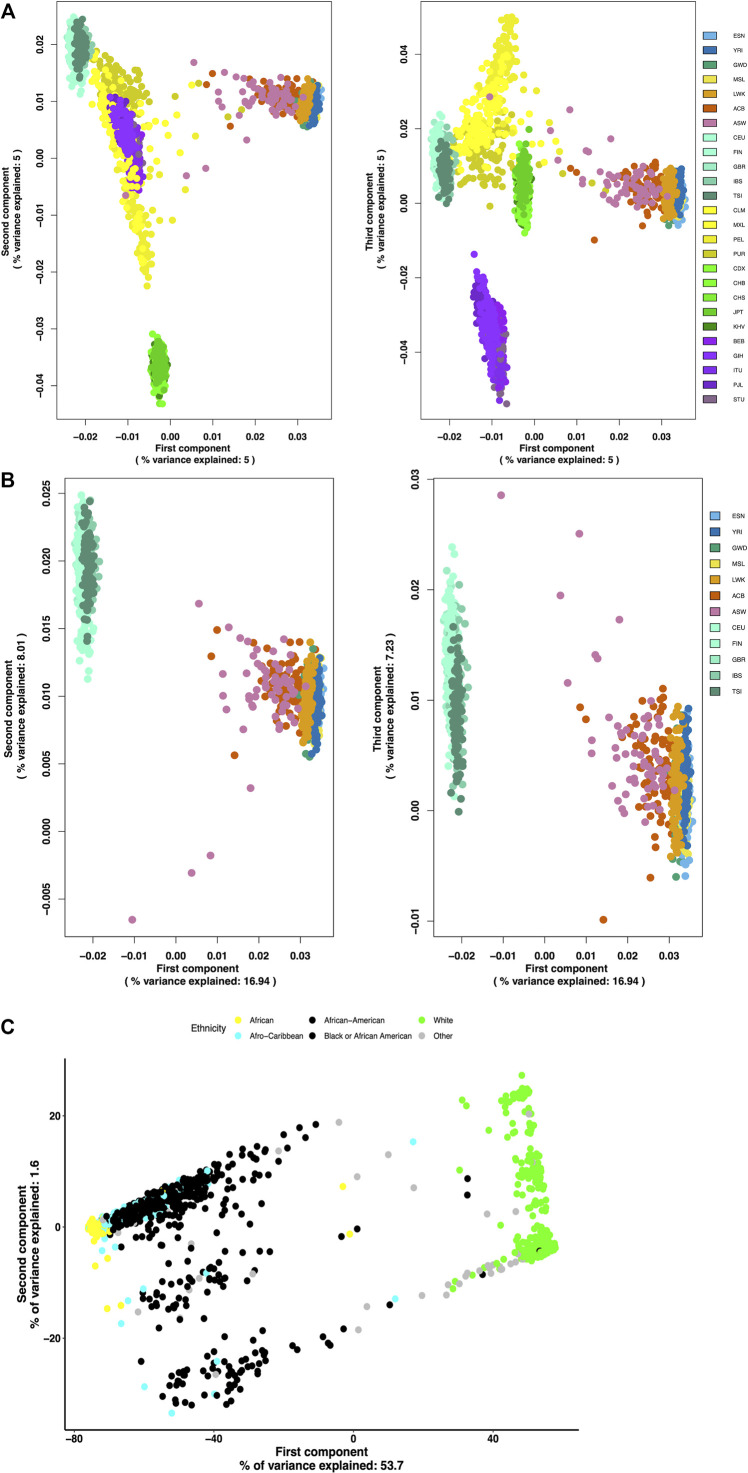
(Continued).

The generated African AIMs were evaluated for their ability to properly differentiate populations of African ancestry from populations of European ancestry and the other continental ancestral groups such East Asia, South Asia, and the Americas. Using SNPs from the 1000 Genomes Project, principal component analysis reveals that this AIMs set separates African subpopulations from all other populations (Figure 4A). Based on these AIMs, the AFR subpopulations are effectively segregated from the EUR subpopulations in addition to other global ancestry groups that were not included in the generation of the set (Figure 4A). The PCA plots also show that the AIMs were able to detect population substructure within populations admixed with African ancestries such as the admixed American populations, ASW and ACB. Figure 4B shows that PC1 separates the AFR subpopulations from the EUR subpopulations and to a lesser extent it shows some degree of separation between the AFR subpopulations, explaining 16.94% of the total variance of the sampled genetic variation. PC2 and PC3 explained a combined additional 15.24% of the total genetic variance. The five continental African (AFR) subpopulations appear to cluster more tightly together while the populations of African ancestry in Southwest United States (ASW) and the African Caribbean in Barbados (ACB) subpopulations show some spread toward the European (EUR) population cluster.

Using an independent dataset of racially self-identified individuals from the United States, we were able to show that our validation subset of 9,385 markers effectively separated individuals of European ancestry from those of African ancestry (Figure 4C). Additionally, these AIMs detected population differences within the group of individuals with African ancestry without being able to fully differentiate the sub-populations of African ancestry (continental African, African American, Caribbean of African ancestry).

### AIM allele frequencies and representation in common genotyping arrays

**FIGURE 5:**
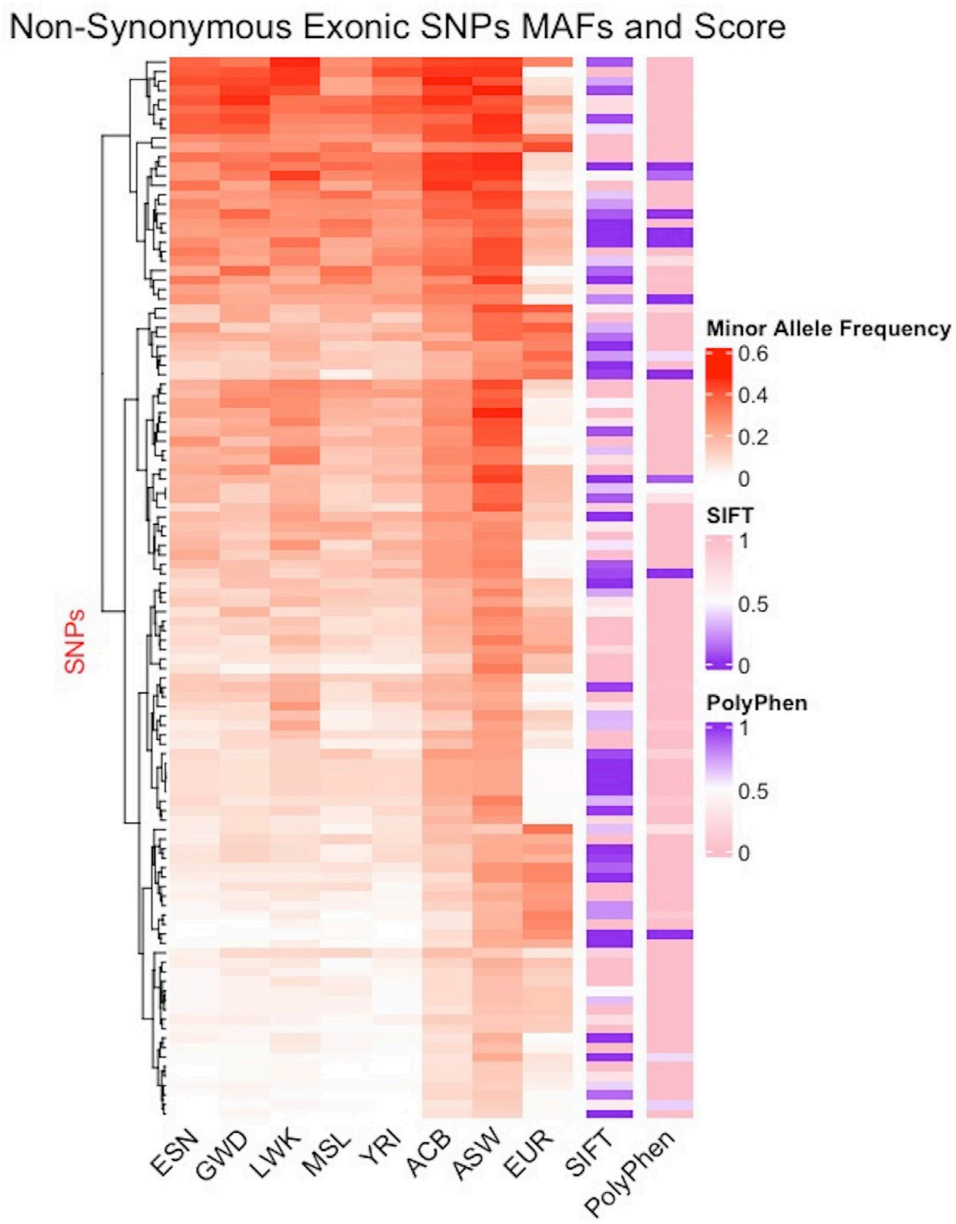
Distribution of minor allele frequencies in African AIMs. AIMs found in the coding regions of the genome and carrying non-synonymous mutations are juxtaposed next to the SIFT and Polyphen2 scores for each SNPs. Of the 338 exonic SNPs, 164 are non-synonymous mutations, 149 of which were scored by the tools. Excluding the X-linked SNPs and multiallelic sites, only 112 SNPs were non-synonymous and are plotted here. The color gradient for the MAF ranges from white (0) to red (0.6); the gradient for SIFT range from purple (0, damaging) to pink (1, benign), while the gradient for Polyphen2 ranges from pink (0, benign) to purple (1, damaging).

Minor allele frequency (MAF) reflects how common an allele is in a population with low frequency alleles often associated with disease phenotypes making them markers of high interest. In Figure 5, columns 1–5 display the MAF for the five continental AFR populations (ESN, GWD, LWK, MSL, YRI), column 6: the Afro-Caribbean population, column 7: African Americans in the Southwest, and column 8: the European population. The five AFR subpopulations appear to have similar MAFs for the different SNPs while the frequencies are noticeably different in the ACB and ASW populations. The EUR populations appear to have much lower MAFs for these SNPs. The seven columns (1–7) with populations of African ancestry show further evidence of population substructure. We also tried to predict pathogenicity using prediction tools such as SIFT (sorting intolerant from tolerant) and PolyPhen2 (polymorphism phenotyping). Column 9 shows the SIFT results and column 10 shows the results from PolyPhen2 with damaging mutations labeled in purple (Figure 5). There are a few instances where some SNPs are identified as damaging by both predictors and these SNPs appear to be more concentrated in SNPs with higher MAFs in the AFR populations. Interestingly, many of the SNPs predicted to be damaging by SIFT have very low MAF in the EUR population. We also compared other pathogenicity tools, such as metaSVM and metalR (Dong et al., 2015), that provide an aggregate prediction score from multiple individual tools and these tools only identified two AIMs as damaging: rs12186491 maps to SPINK6, a serine protease inhibitor kazal-type 6 gene which has been found to regulate nasopharyngeal carcinoma metastasis through EGFR signaling, and rs6601495 encodes for Retinitis Pigmentosa 1-Like 1 Protein which is associated with diseases like occult macular dystrophy and retinitis pigmentosa (Zheng et al., 2017; Noel and MacDonald, 2020).

**FIGURE 6:**
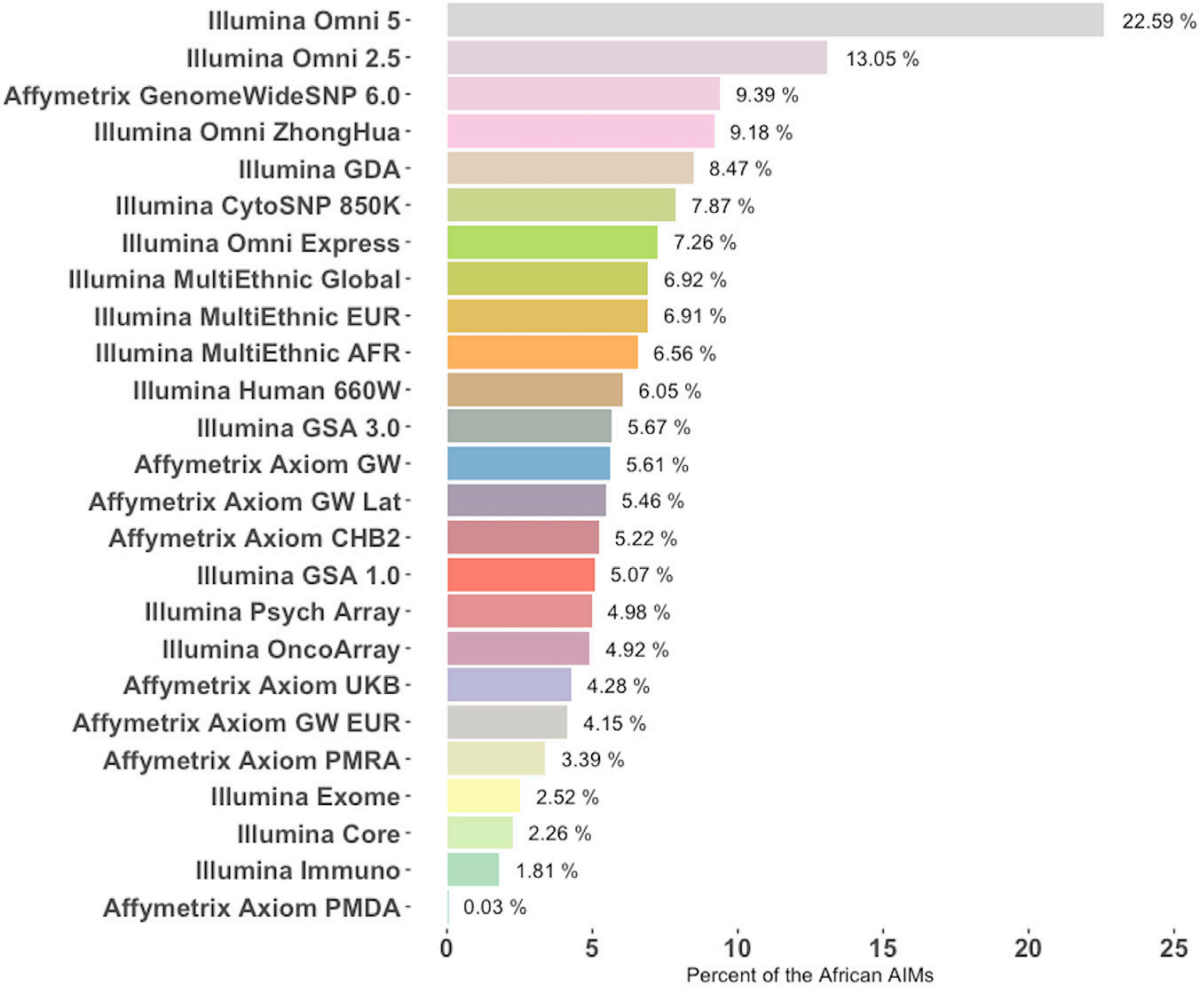
Percentage of African AIMs from this study that are represented in commercial genotyping arrays. Twenty-five commonly used genotyping platforms were chosen from Illumina and Affymetrix (*y*-Axis) and the fraction of SNPs differentiating the African population from others (i.e., AIMs from this study) is listed for each array. Platforms are ranked from most representative of the AIMs panel from this study to the least.

Generally, functional or pathogenicity information on the SNPs contained in this set of AIMs is scarce which may be a byproduct of being underrepresented in currently available genotyping platforms. An analysis of 25 popular genotyping array platforms from Illumina and Affymetrix reveals that most of the commercial arrays surveyed included less than 10% of the African AIMs (Figure 6). Combined, only 19,239 of the 46,737 AIMs in this set were found among the 8,708,293 unique SNPs included in these 25 commercial arrays. When compared to a current list of over 13 million variants (imputed SNPs from Neale lab, https://github.com/Nealelab/UK_Biobank_GWAS) that were imputed against a diverse panel, 43,647 of the 46,737 AIMs were found, indicating that most of the SNPs could be imputed.

## Discussion

Undetected population structure can lead to spurious findings in genetic association studies. With the increased reliance on these studies to identify genetic markers associated with disease, identification and correction for population stratification are critical as both environmental and genetic factors can affect disease risk between populations or subpopulations (Enoch et al., 2006). In this work, we developed a set of African ancestry-informative SNPs that differentiates populations of African ancestry from others and identifies substructure within populations of African ancestry based on estimates of informativeness. We identified 46,737 African ancestry-informative markers from five African subpopulations using Phase 31,000 Genomes Project data and our results suggest they convincingly aggregate populations based on their genetic ancestry and effectively separate populations of African ancestry from other major ancestral populations (Figure 4). Although the AIMs were identified from a comparison between AFR subpopulations and a combined EUR reference, Figure 4 shows that they suitably isolate populations of African ancestry from those of other continental groups including East Asian, South Asian, and groups with American ancestry. While there are several existing sets of ancestry-informative SNPs claiming to differentiate African populations from others, they often come with limitations including that they are estimated from one subpopulation of African ancestry, usually YRI, and one of European ancestry, usually CEU (Keene et al., 2008; Cheng et al., 2009; Tandon et al., 2011; Zeng et al., 2016), though sometimes they include ASW (Chen et al., 2010). This AIM panel provides a more extensive and comprehensive set of pan-African SNPs that can help improve the accuracy of African ancestry estimates since it was established from multiple subpopulations from both the European and African populations.

We developed our AIMs set to exclude SNPs that are specific to just one African subpopulation and are unlikely to be informative outside the context of that subpopulation. We also detected substructure between subpopulations of African ancestry when admixed populations with significant African ancestry, namely ASW and ACB, were introduced (Figure 2). However, this AIMs panel of markers does not fully differentiate between subpopulations of African ancestry. This distinction highlights the consideration that should be given to both between and within population variation to effectively control for population stratification in genetic studies. As advanced in Enoch et al. (2006) and originally by Lewontin (1972), while there is significant diversity between populations, the bulk of human diversity is found within populations.

We evaluated the subset of AIMs that are in protein-coding regions for pathogenicity and association with disease as certain disease-causing variants have been found at highly differing frequencies across populations (Patterson et al., 2004). Using tools such as SIFT, Polyphen, metaSVM, and metaLR *via* ANNOVAR, we investigated which changes may lead to a loss of function in their associated proteins. These tools predict how non-synonymous mutations affect protein function (Flanagan et al., 2010). 0.64% of the AIMs are located in exonic regions of the genome and of that SNP pool, only the 48.52% fraction that are non-synonymous mutations were used for pathogenicity prediction. There is also a lack of agreement between each of the prediction tools which makes it challenging to interpret the predictions made for the SNPs identified as damaging. Further investigation is needed to contextualize these AIMs and elucidate their potential implications in disease.

Although most of our identified AIMs are in non-coding regions, they can still play a role in the genetic basis of health disparities and how genetic ancestry can influence disease risk, although these association data are sparser. One such case of that demonstrates the functional impact of non-coding AIMs from association literature is the African ancestry variant, rs72725854, a rare variant found in an enhancer region at 8q24 which has been shown to regulate multiple lnRNA genes and the MYC oncogene to influence prostate cancer risk in men of African ancestry (Darst et al., 2020; Walavalkar et al., 2020). Additionally, the GWAS literature is ripe with SNPs that have been found to be associated with disease phenotypes such as differential survival in head and neck cancer, differences in prostate cancer risk, and differences in diagnosis stage in breast cancer (Al-Alem et al., 2014; Irizarry-Ramírez et al., 2017; Ramakodi et al., 2017).

Certain chromosomes stood out as having a higher proportion of AIMs relative to their size such as the X-chromosome and chromosomes 4, 8, 12, 14, 15, and 17, all of which have a higher proportion of AIMs than the much larger chromosome 1. Interestingly, loci on some of these chromosomes have been identified in the literature as associated with diseases in populations of African ancestry including loci 8p23 and 8q24 and asthma risk and the association of rs75853687 on chromosome 5 with alloimmunization in sickle cells patients (Williams et al., 2018; Daya et al., 2019). Over eight variants associated with prostate cancer risk have been identified on loci 8q24 (Han et al., 2016).

This AIMs panel will significantly contribute to the ease with which the field integrates African genetic ancestry in population genetics studies but there remain some limitations. The 1000 Genomes Project was used to identify the AIMs for this set although it only includes six African sub-populations and does not completely encapsulate the rich genetic history of the African continent. Moreover, the included populations have small sample sizes which might not be fully representative of the diversity in those populations. Lastly, Phase 3 data from the 1KGP was sampled at relatively low depth (7.4x) which can make it challenging to identify less common, rarer variants in the population (Byrska-Bishop et al., 2022).

The underrepresentation of these African AIMs on commonly used commercial genotyping arrays also contributes to the scarcity of information about their involvement of disease. However, many of these SNPs may be imputable using currently available African-inclusive panels. We searched a list of over 13 million imputable variants generated from UK BioBank data imputed using a panel made up of data from the Haplotype reference consortium, UK10K, as well as the 1000 Genomes Project reference panels, for the presence of our identified African AIMs. Out of these 46,737 AIMs, 43,647 were found on the list of imputable variants, which is expected considering that 1KPG data was included in the panel used to impute the SNPs. However, many populations in Africa remain poorly studied and imputing variants for those could be challenging. As discussed by Martin et al. (2018), imputation panels and resources have a European bias, and sequencing initiatives are biased for West African populations, thus, ignoring much of the total genetic diversity on the continent. This is a particular concern for the imputation of African genomes as the literature suggests a higher rate of genetic diversity and a lower rate of linkage disequilibrium in African populations (Bentley et al., 2020). Furthermore, most reference panels are currently not publicly available or use too small of a sample size to impute effectively (Gurdasani et al., 2015; Mathias et al., 2016; Taliun et al., 2021).

The development of ancestry-informative markers within the framework of population disease risk estimation presents an exciting opportunity to investigate the genetic bases of health disparities across heterogeneous populations with people from different genetic histories. Population structure can have significant implications for genomics studies and simply controlling for them is not always enough to successfully account for population stratification and to avoid such pitfalls as spurious associations (Enoch et al., 2006). As demonstrated here, having a highly specific African AIMs set can help detect ancestry differences between populations of African ancestry and others but it can also identify substructure within populations of African ancestry that should be further surveyed in association studies. This approach increases statistical power and can lead to the identification of true associations between the SNP markers of interest and the disease/trait in association studies. Many studies have identified associations between genetic ancestry and health and, more recently, there is a trend toward the identification of AIMs associated with disease though the studies of African ancestry are still few. Studies like these allow for increased granularity in the analysis of ancestry and health. Considering that self-reported race is an inconsistent and unreliable substitute for genetic ancestry, the AIMs set presented here provides a means for researchers to uncover the impact of ancestry on disease and phenotype. These African AIMs, allowing researchers to apply a set of markers spanning the whole genome, will hopefully provide new avenues to study disease genetics in a large, diverse, and understudied population, and helps elucidate the contribution of local ancestry to disease risk and health.

## Data Availability

Publicly available datasets were analyzed in this study. This data can be found here: 1000 Genomes project. The list of ancestry-informative markers developed in this study is available in Supplementary Table S1.
